# Enhancer of zeste homolog 2 is a negative prognostic biomarker and correlated with immune infiltrates in meningioma

**DOI:** 10.3389/fnins.2022.1076530

**Published:** 2022-11-30

**Authors:** Jing Zeng, Lu Sun, Jiaming Huang, Xia Yang, Wanming Hu

**Affiliations:** ^1^Department of Pathology, Sun Yat-sen University Cancer Center, Guangzhou, China; ^2^State Key Laboratory of Oncology in Southern China, Sun Yat-sen University Cancer Center, Guangzhou, China

**Keywords:** EZH2, meningioma, H3K27me3, GEO, PD-L1, immune infiltrates

## Abstract

**Background:**

Enhancer of zeste homolog 2 (EZH2), an important epigenetic regulator, that mainly regulates histone H3 lysine 27 trimethylation (H3K27me3) through histone methyltransferase, and participates in promoting the development of tumors. At present, the loss of H3K27me3 expression in meningioma is a poor prognostic factor, but the research of EZH2 in meningioma is rare. Therefore, we aim to explore the expression of EZH2 in the meningioma and its correlation with the prognosis and immune microenvironment and lay the foundation for the subsequently potential targeted therapy and immunotherapy for meningioma.

**Methods:**

Tissue microarray immunohistochemistry staining was performed on 276 meningioma samples from Sun Yat-sen University Cancer Center. Expression levels of EZH2, H3K27me3, Ki67, programmed cell death protein 1 (PD-1), programmed cell death 1 ligand 1 (PD-L1), CD4, CD8, CD20, FOXP3, CD68, and CD163 were evaluated. Cox regression analyses were performed, and the Kaplan–Meier (KM) method was used to construct survival curves. In addition, we use biological information methods to analyze the mRNA expression of EZH2 and its relationship with the prognosis and immune microenvironment in the gene expression omnibus (GEO) database.

**Results:**

Enhancer of zeste homolog 2 expression is concentrated in World Health Organization (WHO) grades 2 and 3 meningiomas (8.3+ and 33.3%+). We found that EZH2 expression was associated with a worse prognosis in meningioma (*P* < 0.001), the same results were confirmed in the GEO database (*P* < 0.001). Both EZH2 expression and H3K27me3 deletion (*P* = 0.035) predicted a worse prognosis, but EZH2 has no correlation with H3K27me3 expression. EZH2 expression was closely associated with increased Ki67 index (*P* < 0.001). In addition, EZH2 was associated with the immune microenvironment and positively correlated with PD-L1 expression (*P* < 0.001).

**Conclusion:**

Enhancer of zeste homolog 2 is a new prognostic biomarker in meningioma. It correlates with PD-L1 expression and closely related to tumor immunosuppression. Our research can provide a reference for the potential targeted therapy and immunotherapy of meningioma in the future.

## Introduction

Meningioma is one of the most common primary tumors of the human central nervous system, accounting for 26.2–38.3% of all intracranial tumors, and arises from the arachnoid cap cells of the leptomeninges (Ostrom et al., 2019). According to the 5th edition of the World Health Organization (WHO) classification, meningiomas can be classified into three grades and 15 subtypes. Approximately 80% of meningiomas enrich for WHO grade 1 (such as meningothelial, fibrous, and transitional meningiomas, etc.), and they are considered to be benign meningiomas with benign histological behavior (Louis et al., 2021). The other 20% of meningiomas [such as atypical meningioma (WHO grade 2) and anaplastic (malignant) meningioma (WHO grade 3), etc.] have an aggressive behavior and a much higher recurrence and mortality rate (Dalle Ore et al., 2019). At present, the treatment of malignant meningiomas still relies on traditional surgery combined with radiotherapy (Gousias et al., 2016; Huntoon et al., 2020). More attention should be paid to further exploring the prognostic and treatment-related biomarkers and novel therapeutic approaches in malignant meningiomas.

Enhancer of zeste homolog 2 (EZH2) is a member of the polycomb genes (PcGs) family, a group of important epigenetic regulators that repress transcription (Cao and Zhang, 2004). It is the catalytic subunit of polycomb repressor complex 2 (PRC2), an enzyme that regulates gene expression through H3 lysine 27 trimethylation (H3K27me3) (Viré et al., 2006). The absence of H3K27me3 expression in meningiomas is currently considered to be a poor prognostic factor (Katz et al., 2018; Nassiri et al., 2021), but studies on EZH2 in meningiomas are still rare (Samal et al., 2020). It has been shown that the combination of EZH2 inhibitors and immunotherapy may be effective in tumor therapy such as EZH2 mutant lymphoma (McCabe et al., 2012; Kim and Roberts, 2016). Therefore, it is significant to explore the expression of EZH2, its prognosis value, its relationship with H3K27me3 in meningiomas and the immune microenvironment for subsequent potential targeting and immunotherapy of meningiomas.

## Materials and methods

### Patient samples and clinical data

A total of 286 meningioma samples were included in this retrospective single-center study. All the tissue samples were resected at Sun Yat-sen University Cancer Center (SYSUCC, Guangzhou, China) from June 2008 to June 2018. Follow-up was last done in June 2022. The clinical data sets included gender, age at diagnosis, histopathological diagnosis (Goldbrunner et al., 2016), tumor location, time of death, time to radiographic tumor recurrence, and radiotherapy treatment between surgery and tumor recurrence. Hematoxylin and eosin (H&E) slides were reviewed from each case to confirm the diagnosis of meningioma by two pathologists (an experienced senior neuropathologist and a resident pathologist). The outcomes of each sample depend on tumor recurrence and time to recurrence. Recurrence was defined as tumor growth following the time of operation, and the time to recurrence was determined by calculating the duration from the date of surgery to the first post-operative imaging documenting tumor recurrence. The extent of resection was extracted from the surgeon’s operative report and checked with post-operative magnetic resonance imaging (MRI). All samples were ethically approved for use based on informed consent and the Ethics Committee of SYSUCC.

### Tissue microarray construction and immunohistochemistry

Archived formalin-fixed and paraffin-embedded tissue samples were used for the extraction of two biopsy punches of 2 mm diameter each to construct tissue microarrays with a conventional tissue microarrayer (Beecher Instruments, Sun Prairie, WI, USA). Tumor cylinder extraction was done after evaluation of H&E slides for suitable areas. The newly formed tissue blocks were cut into 4 μm slices with a microtome. After subsequent drying at 80°C for 15 min, subsequent immunohistochemical staining for H3K27me3 (1:200, rabbit monoclonal antibody C36B11, Cell Signaling, Danvers, MA, USA) and EZH2 (1:100, rabbit monoclonal antibody D2C9, Cell Signaling, Danvers, MA, USA) were done with a Ventana BenchMark immunostainer (Ventana Medical Systems, Tucson, AZ, USA). Analysis of immunohistochemical staining were scored independently by two pathologists.

### Immunohistochemical analyses

H3 lysine 27 trimethylation staining was assessed for nuclear expression on tumor cells. At least 1,000 tumor cells were counted under ×400 magnifications starting with the hot spot. The vascular endothelial cells and inflammatory cells were carefully excluded while counting morphologically. We calculate the cutoff value by the receiver operating characteristic (ROC) curve, and the labeling index of EZH2 was further sub-grouped as 0 for no positive cells, 1 for ≤25% of positive cells, and 2 for >25% of positive cells. In the case of heterogeneity of coloring intensity, the intensity of the major proportion of cells was considered. For EZH2, score 1 and 2 were considered low expressions and high expressions, and both score 1/2 were considered positive expressions. The expression of H3K27me3 was scored according to the positive proportion of the tumor cells. The percentage of positively stained tumor cells was scored as follows: 0 (<20% positive tumor cells) (Schaefer et al., 2016), 1 (≥20 and <50% positive tumor cells), and 2 (>50% positive tumor cells). The detailed protocol and assessment standard of other antibody markers including programmed cell death protein 1 (PD-1), programmed cell death 1 ligand 1 (PD-L1), CD4, CD8, CD20, FOXP3, CD68, and CD163 were described in a previous study (Du et al., 2015). We assessed and counted all immunohistochemical profiles of immune cell expression and divided them into two groups of high and low expression using the mean value (Table 1) as the cut-off value. The only difference was PDL1, which we divided it into expression and non-expression (<1%) groups by TPS (tumor cell proportion score), where the expression/positive group was divided into low expression (≥1–49%) and high expression group (≥50%) (Han et al., 2016).

**TABLE 1:** Expression of enhancer of zeste homolog 2 (EZH2), H3 lysine 27 trimethylation (H3K27me3), and clinicopathologic parameters in meningioma.

Parameter	Subcategory	Total	Loss H3 lysine 27 trimethylation	*P*	Enhancer of zeste homolog 2 positive	*P*
*n* (%)	276	276	39 (14.1)		26 (9.4)	
Age	49.58 ± 13.076 (12–86)					
Sex	Male	97 (35.1)	15 (15.5)	0.64	11 (11.3)	0.275
	Female	179 (64.9)	24 (13.4)		15 (8.4)	
WHO grade	1	129 (46.7)	11 (8.5)	0.031	4 (3.1)	< *0*.001
	2	108 (39.1)	19 (17.6)		9 (8.3)	
	3	39 (14.1)	9 (23.1)		13 (33.3)	
Subtype	Meningothelial	76	4 (10.3)		1 (1.3)	
	Transitional	22	2 (5.1)		2 (9.1)	
	Secretory	2	1 (2.6)		0	
	Fibrous	16	1 (2.6)		1 (6.3)	
	Psammomatous	4	0		0	
	Angiomatous	5	2 (5.1)		0	
	Microcystic	4	1 (2.6)		0	
	Atypical	106	19 (48.7)		10 (9.4)	
	Choroid	2	0		0	
	Clear cell	1	0		0	
	Anaplastic	34	8 (20.5)		10 (29.4)	
	Papillary	3	0		2 (66.7)	
	Rhabdoid	1	1 (100)		0	
Location	Skull base	127 (46)	17 (43.6)	0.743	11 (7.4)	0.147
	Not skull base	149 (54)	22 (56.4)		15 (11.8)	
Ki67	< *5*%	120 (43.5)	13 (33.3)	0.168	2 (1.7)	< *0*.001
	≥5%	156 (56.5)	26 (66.7)		24 (15.4)	

*P*-value: Chi-square test.

### Bioinformatic analysis in cancer datasets

The meningioma RNA-seq data were downloaded from https://www.ncbi.nlm.nih.gov/geo/. We analyzed 181 gene expression omnibus (GEO) RNA-seq cohorts of meningioma, ranging from WHO grade 1–3. The meningioma and meningeal RNA-seq data were from GEO datasets, including GSE43290, GSE74385, and GSE16581. Limma package in R software was conducted to screen out the differentially expressed genes (DEGs) with the cut-off criterion of adjusted *P* < 0.05 and | log2FC| > 1. The dataset GSE16581 with survival information we used to perform survival scores, compare the survival differences between high and low expression groups, and Kaplan–Meier (KM) plot survival curves by the Survival package and Survminer package in R. We divided all meningioma data into high and low expression groups based on the cutoff value of the median EZH2 mRNA expression, and compared and analyzed the differences in immune cell expression between high and low expression groups and plotted the graphs. To explore the functions and pathways of related genes, we performed gene ontology (GO) and Kyoto encyclopedia of genes and genomes (KEGG) analyses on ClueGo and Metascape websites.

### Statistical analysis

GraphPad Prism 8 and SPSS 25 software were performed for statistical analyses. The measurement data was represented as mean ± SD. The Chi-square test was conducted to explore the correlations between the expression of EZH2, H3K27me3, and clinicopathological or immune features. Categorical factors between EZH2 groups were compared using Fisher’s exact tests. Survival distributions of groups were compared using KM estimates and log-rank tests. The Cox proportional hazards regression model was used for univariate and multivariate analyses to evaluate the independence of EZH2, H3K27me3, and other markers in predicting prognosis. A *P*-value < 0.05 was considered to be statistically significant.

## Results

### The expression of enhancer of zeste homolog 2 in meningiomas and its correlation with clinicopathological parameters

**FIGURE 1:**
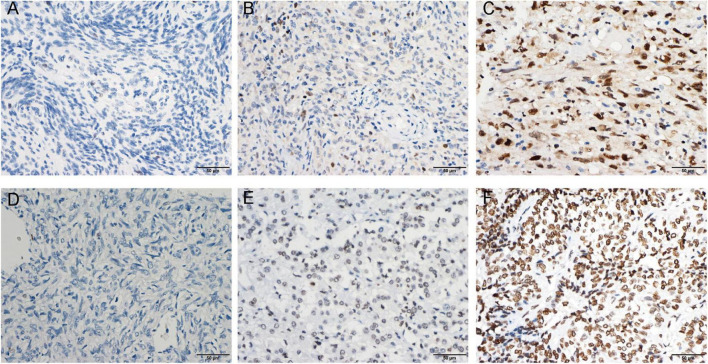
Enhancer of zeste homolog 2 (EZH2) protein expression was detected by immunohistochemistry (IHC). Representative images of negative **(A)**, low expression **(B)**, and high expression **(C)** staining of EZH2 are shown. Representative images of the high density of H3 lysine 27 trimethylation (H3K27me3) **(D)** in loss, moderate **(E)**, and strong **(F)** staining meningioma tissues are shown.

**FIGURE 2:**
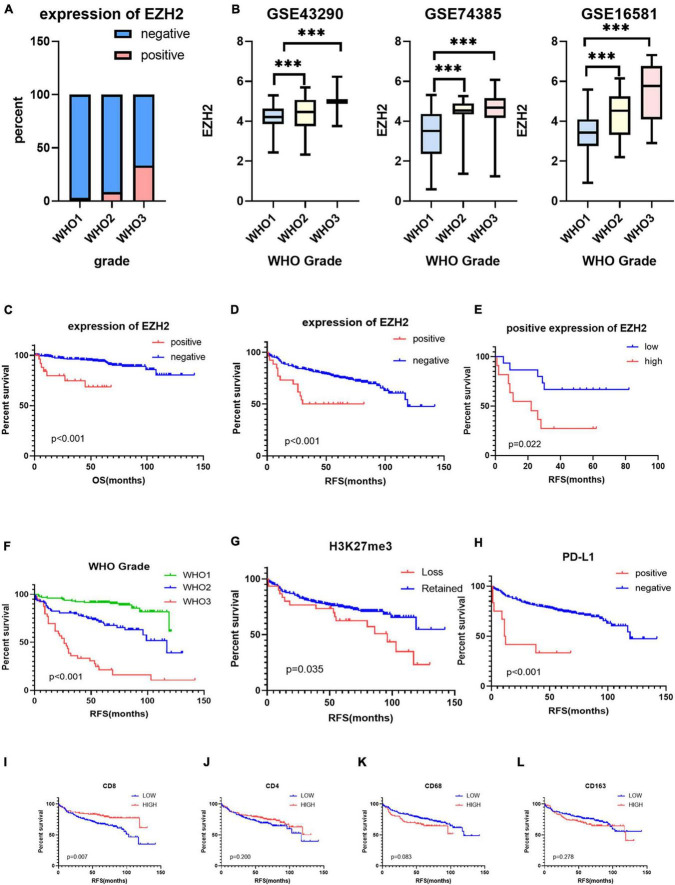
**(A)** The protein expression levels of enhancer of zeste homolog 2 (EZH2) in different World Health Organization (WHO) grades of meningioma. **(B)** The mRNA expression levels of EZH2 in meningioma in gene expression omnibus (GEO) datasets (***P < 0.001). **(C,D)** The prognostic impact of EZH2 expression in meningioma, Kaplan–Meier (KM) curves reveal expression of EZH2 predicts short overall survival (OS) time and short recurrence-free survival (RFS) in meningioma. **(E)** The prognostic impact of EZH2 high expression in meningioma, KM curves reveal expression of EZH2 predicts short RFS in meningioma. **(F–L)** KM survival curves for WHO grade and expression of H3 lysine 27 trimethylation (H3K27me3), programmed cell death 1 ligand 1 (PD-L1), CD4, CD8, CD68, and CD163.

We collected clinical information from 286 meningioma patients and 10 were lost of follow-up. Finally, 276 meningioma patients were included in our retrospective study. Out of the 276 cases, 129 (47%) cases were WHO grade 1, 108 (39%) cases were WHO grade 2, and the remaining 39 (14%) cases were WHO grade 3. The mean age of the patients was 49.58 ± 13.08, with a median age of 50.50, and the majority was the elderly, and only three patients being underage. A total of 179 cases (65%) of patients were women. We also counted Ki67 expression, and based on previous reports in the literature that high Ki67 expression correlates with poor prognosis, we divided the Ki67 expression into low and high expression groups (<5 vs. ≥5%) (Olar et al., 2015) based on immunohistochemical results. In addition, we divided into two groups (Skull base vs. not skull base) according to the location of tumorigenesis. The details were showed in Table 1. By evaluating the expression of EZH2 (Figures 1A–C) and H3K27me3 (Figures 1D,F) by immunohistochemistry, we found some correlations between expression and clinical information. Most H3K27me3 positive cases showed a low level of EZH2 expression, and strong expression of EZH2 was detected in only 11 cases, 4 of which were WHO grade 2 and 6 of which were WHO grade 3 (Table 1 and Figure 2A). EZH2 expression was correlated with increased Ki67 index (*p* < 0.001), for immunohistochemical positive expression was more prominent in areas of high proliferative activity. To further explore the mRNA expression of EZH2 in meningiomas, we analyzed three datasets of meningioma cases from the GEO database and found that mRNA expression of EZH2 was higher in WHO grade 2–3 meningiomas than in WHO grade 1. This is consistent with our results of the immunohistochemical exploration of EZH2 expression (Figure 2B).

### Impact of enhancer of zeste homolog 2 expression on the prognosis of meningioma

**FIGURE 3:**
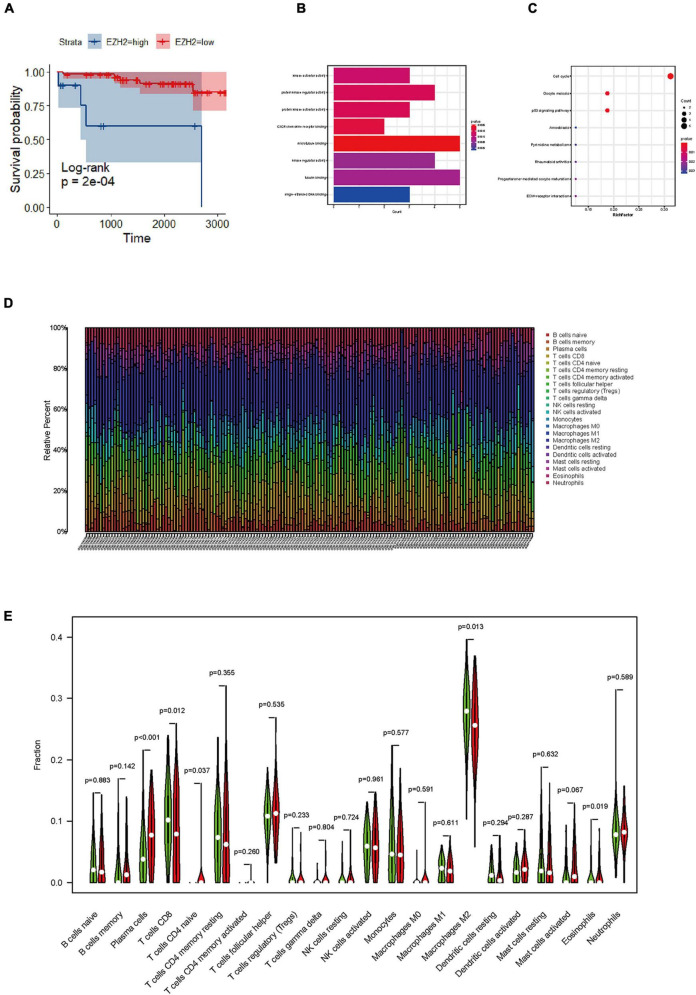
Identification of functions, and pathways in meningioma. **(A)** The prognostic impact of enhancer of zeste homolog 2 (EZH2) mRNA levels in meningioma in gene expression omnibus (GEO) datasets. KM curves reveal high EZH2 mRNA levels predict short overall survival (OS) time in meningioma. **(B)** Gene ontology (GO) analysis shows multiple biological processes of overlapping differentially expressed genes (DEGs). **(C)** Several pathways of overlapping DEGs are identified by Kyoto encyclopedia of genes and genomes (KEGG) analysis. **(D)** The graph of immune cell expression in meningioma after merging data from GEO databases. **(E)** The level of EZH2 mRNA expression correlates with sets of immune cells in meningioma.

The samples included in this cohort showed the expected distribution patterns of recurrence-free survival (RFS) when stratified by WHO grade, with WHO grade 2–3 tumors showed significantly shorter times to recurrence when compared to WHO grade 1 tumors (Figure 2A, log-rank test *P* < 0.001). Mean RFS was 108 months (95% CI 102.5−114.2), 88 months (95% CI 77.6−99.1), and 42 months (95% CI 28.2−56.6) for WHO grade 1–3 tumors (Figure 2F), respectively. We analyzed the survival analysis of two groups of meningioma patients with EZH2 positive and negative, and we found that there was a statistically significant difference in the RFS, demonstrating that expression of EZH2 was associated with poorer RFS in meningioma [Mean RFS 48.9 (95% CI 35.9−62.0) months vs. 102.1 (95% CI 94.1−110.1) months, *P* < 0.001, Figure 2D]. Similarly, we found that there was a worse prognosis in the high EZH2 expression groups than low EZH2 expression groups which divided into two groups by cutoff value (>25 vs. ≤25%) [Mean RFS 26.6 (95% CI 12.9−40.4) months vs. 61.3 (95% CI 46.1−76.4) months, *P* = 0.022, Figure 2E]. In addition, although meningioma patients have a lower mortality rate, we still did overall survival (OS) survival analysis and the result also showed EZH2 expression predicted a worse prognosis (Figure 2C). However, we cannot yet consider it as an independent prognostic factor (Table 2). The dataset GSE16581 in the GEO database contains survival information, and the OS prognostic analysis also showed EZH2 high expression group had a shorter survival time (Figure 3A).

**TABLE 2:** Multivariate analysis of prognostic variables of recurrence-free survival (RFS).

Variables	HR	95% CI	*P*-value
**WHO grade**			
2 vs. 1	1.934	0.965–3.879	0.063
3 vs. 1	4.911	2.214–10.893	<0.001
**H3 lysine 27 trimethylation**			
Retain vs. loss	0.570	0.341–0.952	0.032
**Enhancer of zeste homolog 2**			
Positive vs. negative	1.080	0.537–2.170	0.829
**Programmed cell death 1 ligand 1**			
Positive vs. negative	2.147	0.885–5.205	0.091
**Location**			
Skull base vs. not skull base	0.938	0.593–1.485	0.786
**Sex**			
Male vs. female	1.552	0.992–2.430	0.054
Age	1.015	0.998–1.032	0.085
**MIB-1**			
≥5% vs. <5%	2.093	1.063–4.118	0.033

CI, confidence interval; HR, hazard ratio.

### Both enhancer of zeste homolog 2 expression and H3 lysine 27 trimethylation deletion predicted a worse prognosis, but enhancer of zeste homolog 2 has no correlation with H3 lysine 27 trimethylation expression in meningiomas

We analyzed the survival analysis of two groups of meningioma patients with H3K27me3 loss and retained, we found that there was a statistically significant difference in the RFS, demonstrating that loss of trimethylation was associated with poorer RFS in meningioma (Figure 2G). In addition, we also analyzed the EZH2 expression positive and negative groups and we found a statistically significant difference in RFS, demonstrating that EZH2 positive expression is associated with poorer RFS in meningiomas (Figure 2D). However, there was no connection between EZH2 expression and the deletion of H3K27me3. Only two cases expressed EZH2 with concomitant deletion of H3K27me3, and both were EZH2 low expression.

### Lower CD8 expression and higher programmed cell death 1 ligand 1 expression in the immune microenvironment of meningiomas predicted a worse prognosis

**FIGURE 4:**
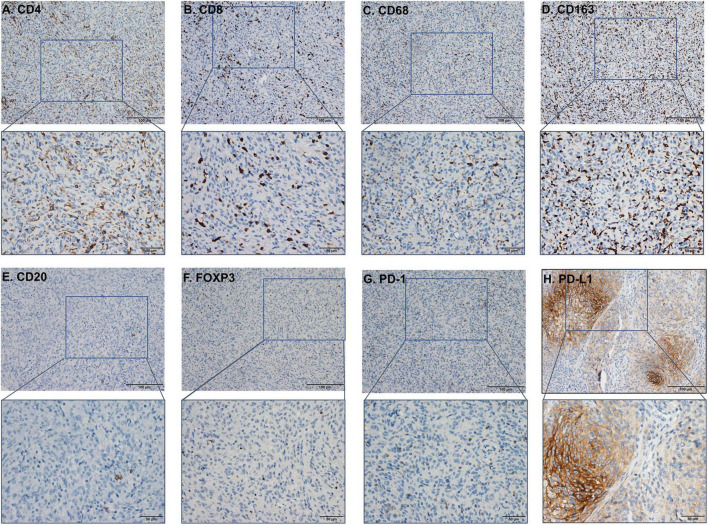
The immunohistochemistry (IHC) figure of various immune cells. **(A)** CD4, **(B)** CD8, **(C)** CD163, **(D)** CD68, **(E)** CD20, **(F)** FOXP3, **(G)** programmed cell death protein 1 (PD-1), and **(H)** PD-L1 (under ×100 and ×400 magnification).

Infiltrating immune cells are important components of the tumor microenvironment and are frequently associated with tumor behavior and patient outcomes. Since several studies and GO analysis revealed that EZH2 was related to the immune response, we further explored the infiltration of immune cells in meningioma (Figure 3D). In addition, we evaluated several immune cell markers, including CD4, CD8, CD20, CD68, CD163, PD-1, PD-L1, and FOXP3 in meningioma samples (Figures 4A–H), and found that the lymphocyte infiltrates in the meningioma tissue was comprised predominantly of T cells with infrequent B cells (Table 3).

**TABLE 3:** Correlation analysis between World Health Organization (WHO) grade and markers of immune cells in meningioma.

Parameter	Mean/mm^2^		Total	WHO 1	WHO 2	WHO 3	*P*-value
CD4	11.43 ± 0.82 (0–75.00)	Low	131 (47.5)	53 (41.1)	54 (50.0)	24 (61.5)	0.065
		High	145 (52.5)	76 (58.9)	54 (50.0)	15 (38.5)	
CD8	16.26 ± 1.16 (0–133.33)	Low	168 (60.9)	65 (50.4)	74 (68.5)	29 (74.4)	0.003
		High	108 (39.1)	64 (49.6)	34 (31.5)	10 (25.6)	
CD163	37.91 ± 1.68 (0–133.33)	Low	173 (62.7)	86 (66.7)	70 (64.8)	17 (43.6)	0.028
		High	103 (37.3)	43 (33.3)	38 (35.2)	22 (56.4)	
CD68	9.62 ± 0.85 (0–83.33)	Low	200 (72.5)	90 (69.8)	85 (78.7)	25 (64.1)	0.139
		High	76 (27.5)	39 (30.2)	23 (21.3)	14 (35.9)	
CD20	0.36 ± 0.12 (0–20.83)	Low	260 (94.2)	122 (94.6)	100 (92.6)	38 (97.4)	0.524
		High	16 (5.8)	7 (5.4)	8 (7.4)	1 (2.6)	
FOXP3	0.06 ± 0.03 (0–4.17)	Low	272 (98.6)	129 (100)	107 (99.1)	36 (92.3)	0.002
		High	4 (1.4)	0	1 (0.9)	3 (7.7)	
Programmed cell death protein 1	0.11 ± 0.04 (0–4.17)	Low	269 (97.5)	124 (96.1)	106 (98.1)	39 (100)	0.340
		High	7 (2.5)	5 (3.9)	2 (1.9)	0	
Programmed cell death 1 ligand 1		Negative	264 (95.7)	128 (99.2)	104 (96.3)	32 (82.1)	<0.001
		Positive	12 (4.3)	1 (0.8)	4 (3.7)	7 (17.9)	

**FIGURE 5:**
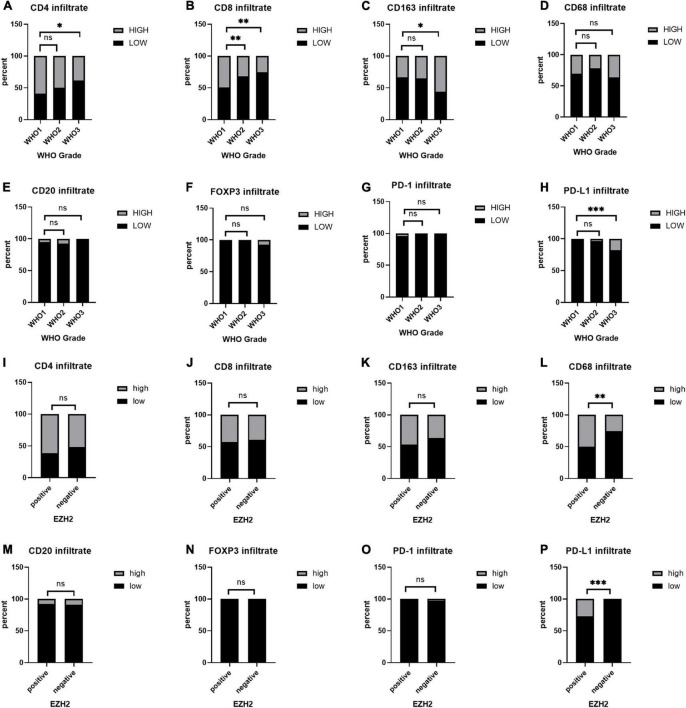
**(A–H)** Immunohistochemical expression of immune cells at different World Health Organization (WHO) levels. **(I–P)** Differences in the expression of immune cell infiltration between the positive and negative groups in enhancer of zeste homolog 2 (EZH2). *P < 0.05, ^**^P < 0.01, and ^***^P < 0.001.

Based on the scores for microarray immunohistochemistry, with a mean cutoff value, we divided the expression of each maker into two groups, high and low expression. We found that the density of immunohistochemical expression of infiltrating lymphocytes was significantly different in WHO grade 2–3 meningiomas compared to WHO grade 1 meningiomas. CD4 and CD8 lymphocytes were all more highly expressed in WHO grade 1 meningiomas, compared to WHO grade 2–3 (Figures 5A–H). This is also consistent with previous studies of reduced T-cell infiltration in WHO grade 2–3 meningiomas. CD20, PD1, and FOXP3 expressions were not found to be different between the different grades of meningioma (Table 3). In addition, we counted the differences in survival analysis between high and low expression of each immune cells (Figures 2J–L) and found that the prognosis of the CD8 low expression group was significantly worse than that of the high expression group (*p* = 0.003; Figure 2I). We further counted the expression pattern of PD-L1+/CD68− and divided into positive and negative groups in this way. Although the overall positive rate of PD-L1 was not high, the PD-L1 positive group was mostly distributed in WHO grade 2–3 meningiomas, especially grade 3 cases, and was statistically significantly different (*p* < 0.001). Survival analyses of the positive and negative groups were counted according to group expression and were found to be statistically significantly different (*p* < 0.001). Positive expression of PD-L1 represented a poorer prognosis (Figure 2H).

### The correlation between enhancer of zeste homolog 2, H3 lysine 27 trimethylation, and markers of immune infiltrates in meningiomas

To study the correlation between EZH2, H3K27me3, and different markers of immune infiltrates in meningioma, we counted the expression of immune cells in the H3K27me3-loss samples and found that the expression of CD8 was lower in the H3K27me3-loss samples compared to H3K27me3-preserved samples. However, there was no significant difference in the expression of CD4, CD20, CD68, CD163, and PD-L1 (Table 4). Interestingly, in the comparison of EZH2 positive and negative, we were surprised to find a significant correlation between EZH2 positive samples and PD-L1 positive samples (<0.001; Figure 5P). In addition, CD68 expression was higher in EZH2-positive specimens compared to EZH2-negative specimens (*p* = 0.010; Figure 5L) and no correlation has been found in other immune cell groups (Figures 5I–K, 5M–O and Table 4).

**TABLE 4:** Correlation analysis between expression of enhancer of zeste homolog 2 (EZH2), H3 lysine 27 trimethylation (H3K27me3), and markers of immune cells in meningioma.

Parameter		H3 lysine 27 trimethylation			Enhancer of zeste homolog 2		
		Loss	Retain	*P*	Positive	Negative	*P*
CD4	Low	24 (61.5)	107 (45.1)	0.057	10 (38.5)	121 (48.4)	0.334
	High	15 (38.5)	130 (54.9)		16 (61.5)	129 (51.6)	
CD8	Low	31 (79.5)	137 (57.8)	0.010	15 (57.7)	153 (61.2)	0.727
	High	8 (20.5)	100 (42.2)		11 (42.3)	97 (38.8)	
CD163	Low	25 (64.1)	148 (62.4)	0.843	14 (51.4)	159 (63.6)	0.328
	High	17 (35.9)	89 (37.6)		12 (46.2)	91 (36.4)	
CD68	Low	30 (76.9)	170 (71.7)	0.501	13 (50.0)	187 (74.8)	0.007
	High	9 (23.1)	67 (28.3)		13 (50.0)	63 (25.2)	
CD20	Low	36 (92.3)	224 (94.5)	0.585	24 (92.3)	236 (94.4)	0.664
	High	3 (7.7)	13 (5.5)		2 (7.7)	24 (5.6)	
FOXP3	Low	37 (94.9)	235 (99.2)	0.097	26 (100)	246 (98.4)	0.672
	High	2 (5.1)	2 (0.8)		0	4 (1.6)	
Programmed cell death protein 1	Low	38 (97.4)	231 (97.5)	0.990	26 (100)	243 (97.2)	0.387
	High	1 (2.6)	6 (2.5)		0	7 (2.8)	
Programmed cell death 1 ligand 1	Negative	38 (97.4.7)	226 (95.4)	0.556	19 (73.1)	245 (99.8)	<0.001
	Positive	1 (2.6)	11 (4.6)		7 (26.9)	5 (0.02)	

To further verify the results of our study, we collected a total of 181 samples from the GEO dataset. We identified overlapping DEGs between EZH2 high expression group and low expression group. The top hub genes were screened *via* the plug-in molecular complex detection and cytoHubba of Cytoscape. GO analysis was performed to show the overlapping DEGs were involved in several biological processes, including kinase activator activity, and CXC chemokine receptors (CXCR) chemokine receptor binding, etc. (Figure 3B). The KEGG pathways are enriched in several classic signaling pathways, such as cell cycle pathway (Figure 3C). We analyzed the difference in the proportion of immune cells according to the gene EZH2 into high and low expression groups and determined the correlation between the expression of the target gene and the content of immune cells. We found that T cells CD4 naive and plasma cells had a higher proportion in the EZH2 high expression group, while T cells CD8 and macrophages M2 had a lower proportion (Figure 3E).

## Discussion

Enhancer of zeste homolog 2 is the catalytic subunit of histone methyltransferase and PRC2. EZH2 catalyzes the H3K27me3 and histone marks, associated with tight chromatin and transcriptional repression, which lead to tumor progression (Kim and Roberts, 2016; Wang et al., 2022). However, there are limited studies on EZH2 expression in meningiomas. We note that Samal explored the prognostic relevance of EZH2 immunohistochemistry in 149 meningioma cases (Samal et al., 2020), but it was limited to WHO grades 1 and 2 meningiomas. Another study enrolled 138 meningiomas and reported EZH2 gene expression was upregulated in atypical samples (Harmancı et al., 2017). In this study, we included 276 meningioma cases of all the grade (129 cases of WHO 1, 108 cases of WHO 2, and 39 cases of WHO 3), enriching as much as possible cases for all tissue subtypes of meningioma. In addition, we screened three datasets through the GEO database to further validate our study. We found that there was a higher EZH2 expression in WHO grade 2–3 meningiomas compared to WHO grade 1 meningioma according to the results of immunohistochemistry. We also further validated higher proportion of EZH2 mRNA in WHO grade 2–3 meningiomas in the GEO database, which is consistent with the immunohistochemical protein results. Moreover, patients with positive EZH2 expression have a shorter time to RFS and a relatively shorter survival time, indicated expression of EZH2 might be a potential prognostic factor.

H3 lysine 27 trimethylation is catalyzed by EZH2, which is involved in the formation of the PRC2 that aggregates to the promoter region and leads to transcriptional repression of the target gene. Methylation of H3K27 is associated with gene repression and plays a notable role in mediating the expression of genes involved in lineage commitment and differentiation (Margueron and Reinberg, 2011). The deletion of H3K27me3 in meningiomas has been considered as an important prognostic factor, but its relationship with EZH2 expression remains unknown. Although the canonical function of EZH2 is gene repression through H3K27 methylation, EZH2 can also act independently of H3K27me3. Hitherto, multiple studies have produced contradictory results. In extranodal NK/T-cell lymphoma (ENKTL), nasal type, aberrant differential expression of EZH2 and H3K27me3 is associated with disease progression and prognosis, EZH2 and H3K27me3 were overexpressed in the majority of ENKTL samples, but EZH2 and H3K27me3 showed an inverse correlation in ENKTL (Liu et al., 2019). In breast cancer, although high EZH2 and low H3K27me3 correlate with poor prognosis of estrogen receptor-positive (ER+) breast cancers, the methyltransferase EZH2 is not required for mammary cancer development (Bae et al., 2015). In mantle cell lymphoma, EZH2 expression is associated with inferior OS and EZH2 expression shows a weak correlation with other PRC2 complex molecules, but no correlation with H3K27me3 expression, or loss of major histocompatibility complex (MHC) I/II (Martinez-Baquero et al., 2021). In contrast, our findings are consistent with the study of mantle cell lymphoma, that there is no correlation between the two markers.

Enhancer of zeste homolog 2 plays a role in the normal biology of many cell types, including immune cells. Dysfunctional EZH2 is associated with the development of multiple cancer types in humans and can promote immune evasion by inhibiting intra-tumoral antigen presentation, immune cell migration, and enhancing CD4+ T regulatory cell (Treg) suppressive activity (Kang et al., 2020; Kim et al., 2020). EZH2 overexpressed tumors often exhibit immunosuppressive tumor microenvironment and immunotherapy resistance (Guo et al., 2020). In prostate cancer, the EZH2 inhibitors combined with PD-1 immunotherapy further improved patient prognosis (Morel et al., 2021). In addition, studies have shown that PD-L1 may be a risk factor for poor prognosis of meningoma, and the PD-1 inhibitor can improve the prognosis of meningoma patients in small samples. This reveals that immunotherapy may be the direction of meningoma therapy in the future. We have investigated the expression of meningiomas with multiple immune-related cells and markers (including PD-1 and PD-L1), and additionally explored their relationship using datasets from the GEO database, and found that high expression of EZH2 have multiple immune-related pathways and associated with several immune cell expression. In prostate cancer, the EZH2 inhibitors combined with PD-1 immunotherapy further improved patient prognosis (Morel et al., 2021). Our study demonstrates that WHO grade 2–3 meningiomas tend to have low CD4, and CD8 expression, which has been confirmed in other studies (Fang et al., 2013; Turner et al., 2022). In previous studies, high CD8 expression was shown to be associated with a better prognosis in meningiomas (Turner et al., 2022). In our study, we also found that high expression of CD8 predicted a better prognosis, but there is no correlation has been found between EZH2 expression and CD8 expression. However, in analysis of immune cell differences in GEO databases, we found that the meningioma EZH2 high expression group had relatively lower CD8 T lymphocytes than the EZH2 low expression group, and it was statistically significant. Interestingly, we found that 12 cases with PD-L1 expression had a worse prognosis, while PD-L1 expression was concentrated in WHO grade 2–3 meningiomas, and PD-L1 expression correlated positively with EZH2-positive cases.

Although we have not been able to demonstrate that immunohistochemical expression of EZH2 in meningiomas correlates with immunosuppression, we found a higher tendency of PD-L1 positivity in EZH2-expressing case. Recently, several studies explored the relationship of EZH2 and PD-L1 in lung cancers. EZH2-expressing lung adenocarcinomas were more frequently to show PD-L1 protein expression than EZH2-negative cases (Toyokawa et al., 2019). Böttcher et al. (2021) found a novel Notch-c-Myc-EZH2 signaling axis which might be controlled PD-L1 upregulation. Zhao et al. (2019) reported EZH2 regulated the immunosuppressive molecule PD-L1 expression *via* HIF-1α in non-small cell lung cancer cells. However, there was no study in meningioma and we will keep studying the potential molecular regulatory mechanism in the future. At present, EZH2 inhibitors including tazemetostat, which has been approved by the Food and Drug Administration (FDA) for use in epithelioid sarcoma (Gounder et al., 2020) and follicular lymphoma (Morschhauser et al., 2020). EZH2 inhibitors in combination with PD-1 inhibitors have been used as a chemotherapy regimen in clinical treatment (Morel et al., 2021). We hold the opinion that the contribution of EZH2 to lymphocyte subpopulation differentiation and function suggested that inhibition of EZH2 has the potential to enhance anti-cancer immunity in certain neoplastic diseases, however, the specific mechanisms involved are yet to be investigated and need to be explored further.

There are still several limitations in our study. First of all, immunohistochemistry is performed by tissue microarray, which restricts our evaluation, for those samples were mainly selected from the central part of the tumor and lacked peripheral tumor tissue. Secondly, our cases were selected before 2018, which lacked molecular testing and markers such as telomerase reverse transcriptase (TERT) and CDKN2A. In addition, our sample size was not large enough.

In conclusion, our study explored the expression of EZH2 and its relationship with H3K27me3 and the immune microenvironment in meningiomas. EZH2 may be a potential prognostic factor and therapeutic target for meningiomas. Our research indicates a direction of EZH2 inhibitors combined with PD-1 immunotherapy for malignant meningiomas in the future.

## Data availability statement

The datasets presented in this study can be found in online repositories RDD (https://www.researchdata.org.cn/), which will be shared by request from any qualified investigator upon approval by the SYSUCC data request committee. The accession number and the data is available from the corresponding authors on reasonable request.

## Ethics statement

The studies involving human participants were reviewed and approved by the Sun Yat-sen University Cancer Center Ethics Committee (B2022-305-01). The patients/participants provided their written informed consent to participate in this study.

## Author contributions

JZ, LS, and WH were designed the study and wrote the manuscript. JH acquired the data. LS and XY were provided help for the IHC test and performed the data analysis. XY, JZ, and WH were reviewed the manuscript. All authors read and approved the final manuscript.

## Abbreviations

CXCR: CXC chemokine receptors
DEGs: differentially expressed genes
EZH2: enhancer of zeste homolog 2
ENKTL: extranodal NK/T-cell lymphoma
ER: estrogen receptor
FDA: food and drug administration
GEO: gene expression omnibus
GO: gene ontology
H3K27me3: histone H3 lysine 27 trimethylation
IHC: immunohistochemistry
KEGG: Kyoto encyclopedia of gene and genomes
KM survival curves: Kaplan–Meier survival curves
MHC I/II: major histocompatibility complex I/II
PD-1: programmed cell death protein 1
PD-L1: programmed cell death 1 ligand 1
ROC curve: receiver operating characteristic curve
TERT: telomerase reverse transcriptase
WHO: World Health Organization.
